# Rutin-Mediated Priming of Plant Resistance to Three Bacterial Pathogens Initiating the Early SA Signal Pathway

**DOI:** 10.1371/journal.pone.0146910

**Published:** 2016-01-11

**Authors:** Wei Yang, Xiaonan Xu, Yang Li, Yingzi Wang, Ming Li, Yong Wang, Xinhua Ding, Zhaohui Chu

**Affiliations:** 1 State Key Laboratory of Crop Biology, Shandong Provincial Key Laboratory of Agricultural Microbiology, Shandong Agricultural University, Taian 271018, China; 2 Institute of Plant Protection, Yantai Academy of Agricultural Science, Yantai 265500, Shandong, China; 3 Tianjin Entry-Exit Inspection and Quarantine Bureau, Tianjin 300300, China; The Chinese University of Hong Kong, HONG KONG

## Abstract

Flavonoids are ubiquitous in the plant kingdom and have many diverse functions, including UV protection, auxin transport inhibition, allelopathy, flower coloring and insect resistance. Here we show that rutin, a proud member of the flavonoid family, could be functional as an activator to improve plant disease resistances. Three plant species pretreated with 2 mM rutin were found to enhance resistance to *Xanthomonas oryzae* pv. *oryzae*, *Ralstonia solanacearum*, and *Pseudomonas syringae* pv. *tomato* strain DC3000 in rice, tobacco and *Arabidopsis thaliana* respectively. While they were normally propagated on the cultural medium supplemented with 2 mM rutin for those pathogenic bacteria. The enhanced resistance was associated with primed expression of several pathogenesis-related genes. We also demonstrated that the rutin-mediated priming resistance was attenuated in *npr1*, *eds1*, *eds5*, *pad4-1*, *ndr1* mutants, and *Nah*G transgenic *Arabidopsis* plant, while not in either *snc1-11*, *ein2-5* or *jar1* mutants. We concluded that the rutin-priming defense signal was modulated by the salicylic acid (SA)-dependent pathway from an early stage upstream of NDR1 and EDS1.

## Introduction

Flavonoids belong to an important class of secondary metabolites in plants, which can be divided into several subgroups by the diversity of chemical radical groups [1]. They exhibit broad biological functions including defense (antibacterial activity), UV protection, auxin transport inhibition, allelopathy, energy transfer, control of respiration and photosynthesis and flower coloring in plant [2]. Rutin is one of the huge families of flavoniods which was broadly distributed in fruits, vegetables and other plant food sources [3, 4]. Even in tobacco leaves, the content of rutin is approximately reached to 80 μg g^-1^ fresh weight [5]. Rutin has anti-inflammatory and strong antioxidant properties too. It was reported to attach to metal ions and prevent reactions that form free radicals [6], to maintain the level of collagen in the skin which prevents signs of aging [7]. In addition, rutin have good anti-diabetic activity on type II diabetic rats [8], and it is potentially an excellent source of functional antihypertensive product which has an inhibitory effect on angiotensin-converting enzyme activity [9]. However, it was not clear whether it could improve itself immunity system in plant.

Plants can acquire enhanced resistance to pathogens after treatment with quiet a few so-called activators such as salicylic acid (SA) and its synthetic analogs, 2,6-dichloroisonicotinic acid (INA), benzo (1,2,3) thiadiazole-7-carbothioic acid (BTH), chitin, β-aminobutyric acid (BABA), lipopolysaccharide (LPS), azelaic acid, hexanoic acid, Vitamin B1 (thiamine), Vitamin B2 (riboflavin) and many other natural or synthetic compounds [10–15]. They will result as activating or priming activation of various cellular defense responses in plants, included early reactive oxygen species (ROS) burst, rapidly induced ion transport changes at the plasma -membrane, callose deposition, the synthesis and secretion of phytoalexins, and the accumulation of transcripts for various pathogenesis-related (*PR*) genes [10, 16]. Most of those activation defense responses were identified to generally dependent on some known resistance signaling pathways, such as systemic acquired resistance (SAR) and induced systemic resistance (ISR) pathways, which use endogenous hormone salicylic acid (SA) and jasmonic acid (JA) as the signal transduction molecules, respectively. However, the activated plant defense by β-aminobutyric acid (BABA) was reported to mediate another signaling mechanism that differs from SAR and ISR [17].

Recently studies were shown that flavonoids are also involved in plant protection. *In vivo* a multitude of studies have revealed a structure-related activity with respect to the anti-fungal effect of flavonoids [1]. For instance, *Pyricularia oryzae*, a fungal pathogen causing blast in rice, has demonstrated a differential sensitivity of growth inhibition against naringenin, kaempferol, quercetin and dihydroquercetin [18]. Including rutin, flavonoids were also found to moderately effective in inhibiting aerobic bacteria causing plant disease [19, 20]. Otherwise, flavonoids were reported to involve in plant immunity as well. A nonsense mutation in the structural gene encoding dihydroflavonol reductase was reported to accumulate the small amounts of dihydroquercetin as well as repress the hyphae penetration for *Fusarium* on testa layers of barley [21]. Spraying quercetin was also identified to prime defense against virulent strain *Pseudomonas syringae* pv. *tomato* DC3000 (*Pst*) in *Arabidopsis thaliana*. It was mediated by H_2_O_2_ burst and the SA signal pathway [22].

Previously study demonstrated that the overexpression of a member of MYB transcriptional factor, the *AtMYB12* gene, resulted in accumulation of flavonoids, especially for the component of rutin in tobacco [5, 23]. The transgenic tobacco was also identified to enhance the resistance to *Ralstonia solanacearum* SD as well as strongly activated many defense-related genes post pathogen inoculation. These results has been borne in mind that rutin might activated the plant immunity directly. In this study, we demonstrated that rutin was acted as a general elicitor which could enhance resistance against bacteria in different hosts, such as rice against *Xanthomonas oryzae* pv. *oryzae* causing rice bacterial blight, *Nicotiana benthamiana* against *Ralstonia solanacearum* SD causing tobacco bacterial wilt and *Arabidopsis thaliana* against *Pst* DC3000. The expressions of *PR* genes were investigated. We also manifested that the rutin-priming defense was modulated by the SA-dependent signal pathway, and it was initiated upstream of the NDR1 and EDS

## Materials and Methods

### Pathogen culture

Bacterial strains *Xanthomonas oryzae* pv. *oryzae* PXO99, *Xanthomonas oryzae* pv.*oryzicola* RH3 were first grown in potato dextrose agar (PSA) liquid medium at the early logarithmic phase. Cells were collected by centrifugation and resuspended in distilled water to form a gradient concentration of 10^6^, 10^7^ and 10^8^ CFU ml^-1^(equal to approximate 0.2 OD). The bacterial suspensions were grown on PSA solid medium containing 0 to 4 mM rutin which purchased from Sangon Biotech (Shanghai, CN), at 28°C for 24 h.

Pathogenic bacteria *Ralstonia solanacearum* SD (Isolated from Shandong Province, China at the year of 2011) and *Pseudomonas syringae* pv. *tomato* DC3000 (*Pst* DC3000) were cultured as described above and the medium were replaced with Nutrient Agar (NA) medium and King’s B (KB) medium, respectively. All the pathogens were only studied in the lab and greenhouse. There has no specific permissions were required for these locations/activities.

### Plant material and pathogen inoculation

Rice Mudanjiang 8 (*Oryzae sativa* cv. japonica) plants were grown in the greenhouse at 28°C, 70% relative humidity and with a 12 h photoperiod. Each of five plants at booting stage were sprayed with a solution containing different concentrations of rutin diluted in distilled water, which was supplemented with 0.02% Tween 20. The control plants were sprayed with 0.02% Tween 20 only. The plants were inoculated with PXO99 (Philippine race 6) by the leaf clipping method after three days of pre-spraying with rutin, as described previously [24]. The disease was scored by measuring the lesion length at 7 and 14 days after inoculation. The results show average values of triple experiments.

*N*. *benthamiana* were grown in the greenhouse under a 16 h light/8 h dark cycle at 25°C, with 70% relative humidity. Eight-week-old plants were sprayed with different concentrations of rutin diluted in distilled water containing with 0.02% Tween 20. The control plants were sprayed with 0.02% Tween 20. The plants were inoculated with 10^8^ CFU ml^-1^ of *R*. *solanacearum* SD through hypodermic injection with a syringe after three days of treatment [25]. Different growth stages of *R*. *solanacearum* SD were detected after inoculation to draw the growth curve. Bacteria in leaves was counted by determining the CFU of 1 g leaves (fresh weight) either pretreated or untreated with rutin on NA medium [20]. At least three plants for each time point were inoculated through leaf injection with the bacterial suspension. The same experiment was repeated in triplicate at the greenhouse.

Seeds of the Columbia (Col-0) ecotype, *npr1-1*(CS3726), *jar1-1*(CS8072), and *pad4-1*(CS3806) were obtained from the *Arabidopsis* Information Resource (http://www.arabidopsis.org). *NahG*, *snc1-11*, *eds5*, *ein2-5*, *eds1*, *ndr1-1* mutants were kindly provided by other contributors. Seeds were chilled at 4°C for 3 days and sown in 50 cm^3^ pots containing a mixture of vermiculite and potting soil. *Arabidopsis* was incubated in a growth chamber with 16 h (200 μmol/m^2^/s) of illumination daily at 20°C and 70–90% relative humidity for 4 weeks before treatments and/or *Pst* DC3000 inoculation. *Pst* DC3000 was stored at -80°C, and cultured on KB medium incubated with 50 μg/ml rifampicin (Rif) at 28°C for 36 to 48 h, adjusting the concentration to 10^8^ CFU ml^-1^ for spray inoculation. The method of rutin treatment is the same as above. The pathogen inoculation is as described previously [26].

### RNA extraction and qRT-PCR

Total RNA was isolated from 100 mg plant tissue with TRI reagent according to the manufacturer’s instructions (T9424, Sigma-Aldrich, USA). 0.5 μg RNA was used for first-strand cDNA synthesis using the PrimeScript^™^ RT reagent Kit with gDNA Eraser (TaKaRa, Dalian, CN). Quantitative PCR was performed with SYBR ^®^ *Premix Ex Taq*^™^ (Tli RNaseH Plus) (Takara, Dalian, CN) on the IQ5 Real-Time PCR System (Bio-Rad, USA). The following PCR program from the reference was used [24]: 95°C for 5 min, followed by 40 cycles of 95°C for 15 s, 55°C for 15 s, and 72°C for 30 s. A heat dissociation curve (55–95°C) following the final cycle of the PCR was checked to test the specificity of the PCR amplification. *OsActin* of rice, *NbEF1α* of tobacco and *AtActin2* of *Arabidopsis* were used as internal control to standardize the results. We used NCBI database to get the gene sequences and Primer Premier 5 to design the primers. For each gene, qRT-PCR assays were repeated at least twice with triplicates runs. Relative expression levels were measured using the 2^-⊿⊿Ct^ analysis method. The sequence of each primer for all detected genes is listed in Table 1.

**Table 1:** Primers used for gene expression analyses.

Primer name	Access number (ID)	Sequence (5’-3’)
Os*PR1a-*F	AJ278436	CGTCTTCATCACCTGCAACTACTC
Os*PR1a-*R		CATGCATAAACACGTAGCATAGCA
Os*PR1b-*F	U89895	GGCAACTTCGTCGGACAGA
Os*PR1b-*R		CCGTGGACCTGTTTACATTTTCA
Os*PR10-*F	D38170	CCCTGCCGAATACGCCTAA
Os*PR10-*R		CTCAAACGCCACGAGAATTTG
Os*LOX-*F	D14000	GCATCCCCAACAGCACATC
Os*LOX-*R		AATAAAGATTTGGGAGTGACATATTGG
*OsCAT-*F	AB020502	CCGCGAGAAGGTGGTGATT
*OsCAT-*R		GTCAGAGAGTGCGTCGATCCA
*OsPOX-*F	X66125	GCCATCATGGACGGTTCTGT
*OsPOX-*R		TCTGGAGAAATTGCCGATAAGTTC
O*sPAL-*F	X87946	AGCACATCTTGGAGGGAAGCT
Os*PAL-*R		GCGCGGATAACCTCAATTTG
OsActin-F	X15865	TGTATGCCAGTGGTCGTACCA
OsActin-R		CCAGCAAGGTCGAGACGAA
Nb*rbohB*-F	AB079499	GTGATGCTCGTTCTGCTCTT
Nb*rbohB*-R		CTTTAGCCTCAGGGTGGTTG
*NbNOA1*-F	AB303300	CCCACTCTTGCTCCTCAAAG
Nb*NOA1*-R		CTGCTTCTTCAGTAGGCACC
Nb*PR1a*-F	X06930	CGTTGAGATGTGGGTCAATG
Nb*PR1a*-R		CCTAGCACATCCAACACGAA
Nb*EF-1α-*F	AY206004	CCTCAAGAAGGTTGGATACAAC
Nb*EF-1α-*R		TCTTGGGCTCATTAATCTGGTC
At*PR1*-F	AT2G14610	GTGCCAAAGTGAGGTGTAACAA
At*PR1-*R		CGTGTGTATGCATGATCACATC
At*PR2*-F	AT3G57260	CTTAGCCTCACCACCAATGTTG
At*PR2*-R		TCCCGTAGCATACTCCGATTTG
At*PR5*-F	AT1G75040	ATGTGAGCCTCGTAGATGGTTAC
At*PR5*-R		GATCCATGACCTTAAGCATGTCG
At*PAL*-F	AT2G37040	TGTAGCGCAACGTACC
At*PAL*-R		GTTCGGGATAGCCGATG
At*ACTIN2*-F	AT3G18780	CTCTCCCGCTATGTATGTCGC
At*ACTIN2*-R		GAAACCCTCGTAGATTGGCA

### Data treatment

All experiments were performed in three replicates with similar results. Each replicate contained at least three plants. The quantitative data were performed by Student’s t test (two-tail *t* test with equal variances; Microsoft Excel) to evaluate the significance in differences between CK and other individuals or treatments. (P < 0.05) are indicated by an asterisk or (P < 0.01) are indicated by two asterisks.

## Results

### Rutin has limited antibacterial action to all tested bacterial pathogens at 2 mM or lower concentration

**Fig 1:**
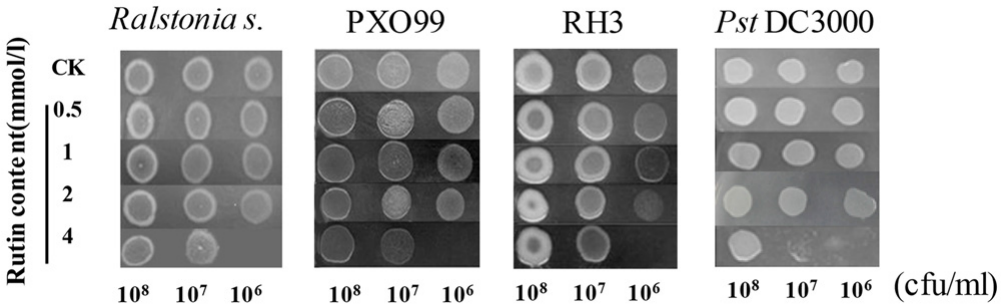
The influence of different consistence of rutin on bacterial pathogens. *Ralstonia solanacearum* SD was cultured on NA medium; *Xanthomonas oryzae pv*. *oryzae* PXO99 and *Xanthomonas campestris pv*. *oryzicola* RH3 were cultured on PSA medium and *Pst* DC3000 was cultured on KB medium with 50 mg/L rifampicin (Rif). The photographs were taken after 24 h incubation at 28°C.

To evaluate the effects of rutin against bacterial pathogens, four strains represented as three bacteria species were growth on cultural medium plus with different concentration of rutin. Among four strains, PXO99 and RH3 were belong to typical strain of *Xanthomonas oryzae* pv. *oryzae* (Xoo) and *Xanthomonas oryzae* pv. *oryzicola* (Xoc), they were shown no clear growth inhibition on PSA contains either 0.5 mM, 1.0 mM or 2mM rutin as well as *R*. *Solanacearum* SD and *Pst* DC3000 (Fig 1). The growth inhibition of all four pathogens was only observed with 4 mM rutin supplemented in PSA when their inoculum titrations were lower as 10^6^ CFU ml^-1^. Compared with rutin, the quercetin demonstrated a better growth inhibition capacity to all four tested pathogens (S1 Fig). These results were indicative of limited antibacterial ability of rutin for tested bacteria.

### Rutin promoted resistance against *Ralstonia solanacearum* in *Nicotiana benthamiana*

**Fig 2:**
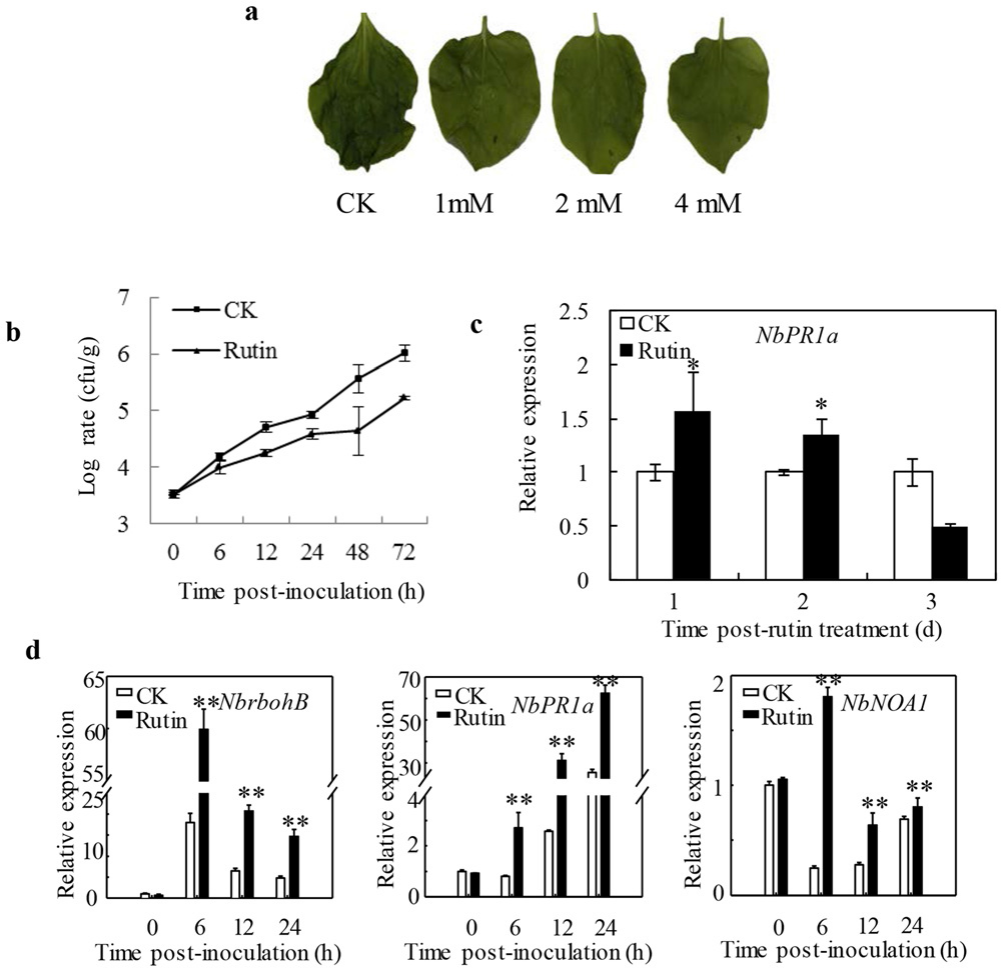
Enhanced to resistant against *Ralstonia solanacearum* SD pretreated with rutin in *Nicotiana benthamiana*. **a** The typical symptoms of *N*.*benthamiana* plants infected by *R*. *solanacearum* SD. The photographs were taken 3 days after inoculation; **b** The growth curve of *R*. *solanacearum* SD in *N*. *benthamiana*; **c** Rutin hardly induced the expression of *NbPR1a* in *N*. *benthamiana*. Samples were collected 1, 2 and 3 days post-treatment with rutin. The values are means ±SE; **d** The expression of three pathogenesis-related genes *RbohB*, *PR1a* and *NOA1* were examined in the primed pattern. Samples were collected from 10 plants at 0, 6, 12 and 24 h post inoculation. The values are means ±SE.

Previous studies described that AtMYB12-overexpressing tobacco was resistant against *R*. *solanacearum* SD as well as enriched rutin. To test whether rutin could directly activated the plant resistance, in this study, we investigated the effect of rutin on the defense response against *R*. *solanacearum* SD in *N*. *benthamiana*. Most leaves in the control group showed water-soaked symptoms, wilted post three days inoculation, as shown in Fig 2a. However, the wilted symptoms were attenuated in *N*. *benthamiana* leaves when it was pretreated with rutin from 1 mM to 4 mM. The stronger attenuation disease symptoms were observed to associate with rutin concentration on inoculation plants (Fig 2a). In addition, rutin hardly inhibit bacteria growth at a concentration of 2 mM in the cultural medium (Fig 1). Therefore, this concentration was chosen for subsequent experiments. The bacterial growth curve indicated that pre-sprayed rutin could remarkably protect *N*. *benthamiana* from *R*. *solanacearum* SD infection at a concentration of 2 mM (Fig 2b). Compared to pretreatment with 2 mM rutin, more than 4.82 folds of bacteria had been evaluated on control plant at 48 hpi (Fig 2b). Additionally, in spite of less antibacterial ability than quercetin, pretreated with 2 mM rutin presented better resistance to *R*. *solanacearum* SD (S2a Fig).

We also analyzed the transcription level of *PR* genes: *NbPR1a*, *NbNOA1* (nitric oxide-associated 1) which is related to NO production and to defense responses [27], and *NbrbohB* (respiratory burst oxidase homologs B) which is related in active oxygen species generation [28]. Without inoculation of *R*. *Solanacearum* SD, the transcription level of *NbPR1a* was slightly up-regulated one day after spraying with rutin and turn to down-regulation at 3 dpi compared with spraying with water in *N*. *benthamiana* (Fig 2c). So we selected three days as the interval time between spraying rutin and *R*. *solanacearum* SD inoculation to balance the weak activation defense caused by spraying rutin. We observed there was a more rapid and strong increased expression levels of *PR* genes, including *NbPR1a*, *NbNOA1* and *NbrbohB* in rutin-pretreated plants than in control plants when *R*. *solanacearum* SD was inoculated (Fig 2d). The transcript levels reached their maximum value at 6 hpi for *NbNOA1* (7.26-fold highter than the control) and *NbrbohB* (3.33-fold highter than the control), and 24 hpi for *NbPR1a* (2.44-fold highter than the control) in rutin-pretreated leaves, respectively. These results suggested that rutin primed the expressing activation of several *PR* genes in challenged *N*. *benthamiana*.

### Suppressed the proliferation of *Xanthomonas oryzae* pv. *oryzae* by pre-spaying rutin on rice

**Fig 3:**
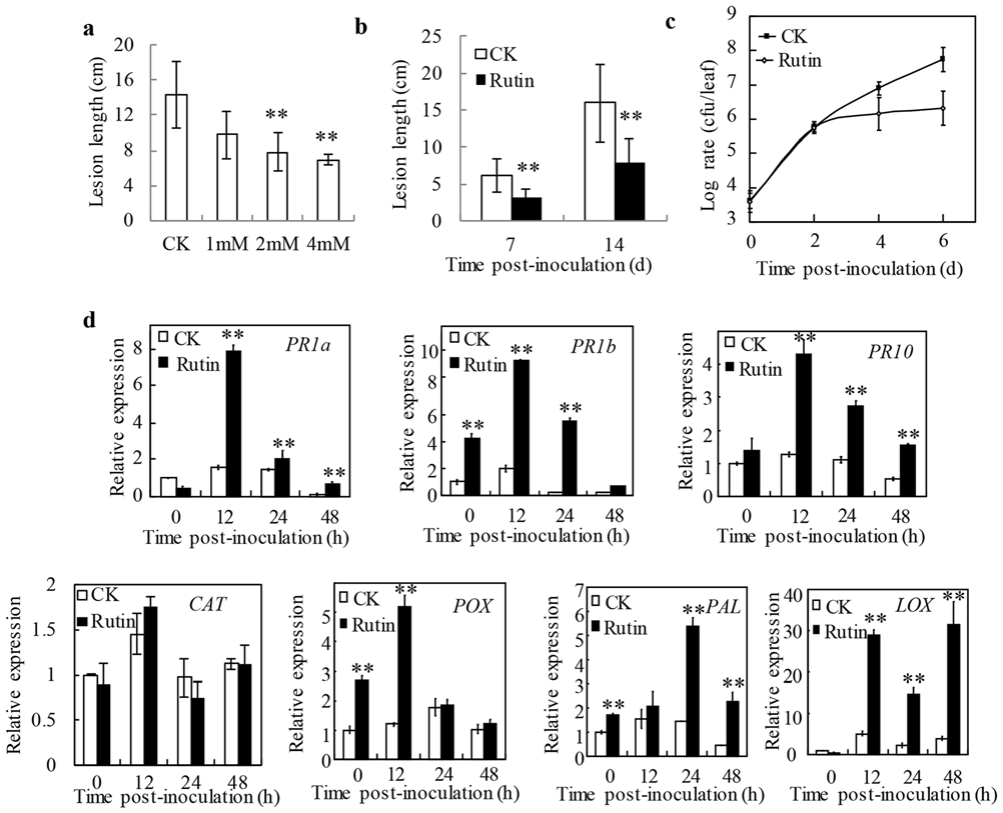
Rutin induced a defense response against *Xanthomonas oryzae* in rice. **a** The measure of lession length causing by *Xanthomonas oryzae* strain PXO99 after rutin pretreatment. The data were collected at 14 days post inoculation. The data represent the mean ±SE of 5 plants; **b** Measured the lesions caused by PXO99 in the rice cultivar MDJ8 by pre-spraying 2 mM rutin. The data represent the mean ±SE of 5 plants; **c** Quantification of PXO99 populations in the rice cultivar MDJ8. Samples were collected from 10 leaves at 0, 2, 4 and 6 days post inoculation. The values are means ±SE; **d** The expression pattern of seven resistance-related genes (*PR1a*, *PR1b*, *PR10*, *CAT*, *POX*, *PAL*, *LOX*) in control and 2 mM rutin-treated rice respectively. Samples were collected from 10 leaves at 0, 12, 24 and 48 hours post inoculation. The values are means ±SE.

To test whether rutin could enhance resistance against bacterial pathogen in other host, we have evaluated the efficacy of rutin against PXO99 which caused bacterial blight disease in rice. The plants were inoculated with PXO99 three days later after sprayed with different concentrations of rutin as 1 mM, 2mM and 4 mM respectively. The lesion length of rice leaves was measured post 14 days inoculation. It was averaged to 14.32 ± 3.75 cm for control plant which pre-spraying with 0.02% Tween 20 only. And the average lesion length was reduced to 9.76 ± 2.65 cm, 7.79 ± 2.19 cm and 6.94±0.57 cm for pretreatment with 1 mM, 2mM and 4 mM of rutin respectively (Fig 3a). Statistical data also suggested that the lesions caused by PXO99 were suppressed in rutin-pretreated Mudanjiang 8 (Fig 3a). As 2 mM rutin inhibited little or nothing to PXO99 *in vitro*, and it has dramatically reduced the lesion length in rice after pre-spraying 2 mM rutin (Fig 1), therefore, we chose 2 mM rutin for subsequent experiments. Capture our attentions, compared with the control, the lesion length was dramatically reduced for rutin pre-spraying rice leaves since 7 d-post-inoculation (Fig 3b). Interestingly, the reductive lesion length was almost similar with each other between rutin- and quercetin- pretreated leaves at 14 dpi (S2b Fig). To investigate whether the pre-spraying 2 mM rutin affected the proliferation of PXO99 in rice leaves or not, we conducted a growth curve experiments in rice. Compared to spray 0.02% Tween 20 only, the number of colonies from spraying rutin leaves has no clear difference post 2 days inoculation, while it was regarding to 5.01 times and 26.92 times reduction at 4 dpi and 6 dpi, respectively (Fig 3c). These results were suggested that pretreated with rutin could enhance rice to against PXO99.

Because enhanced plant disease resistance is usually related to the expression of *PR* genes, to elucidate the rutin-mediated resistance, the expression pattern of several *PR* genes was investigated in rice. The results demonstrated that all six *PR* genes included *PR-1a*, *PR-1b*, *PR-10*, phenylalanine ammonia lyase (*PAL*), peroxidase (*POX*) and *LOX* genes were up-regulated expression after inoculation with PXO99 both for pre-spraying with 0.02% Tween 20 and 2 mM rutin. But the expression of chloramphenicol acetyl transferase (*CAT*) was not significant changed post rutin treatment compared to the control. (Fig 3d). The expression levels of *PR-1a*, *PR-1b PR-10* and *POX* genes were all reached their maximum levels at the 12 h post inoculation, and were approximately 4.98-, 5.05-, 3.39- fold and 4.23-fold higher than the control, respectively. The maximum transcription level of the *PAL* gene was obtained at 24 h post inoculation. The transcription of *LOX* was also induced more highly in treated plants compared to the control plants after inoculation. It was reached high values at the 12 h and 48 h time points after the initiation of inoculation which was approximately 5.71- and 8.23-folds higher than the control plants, respectively.

### Enhanced the resistance against to *Pst* DC3000 in *Arabidopsis thaliana*

**Fig 4:**
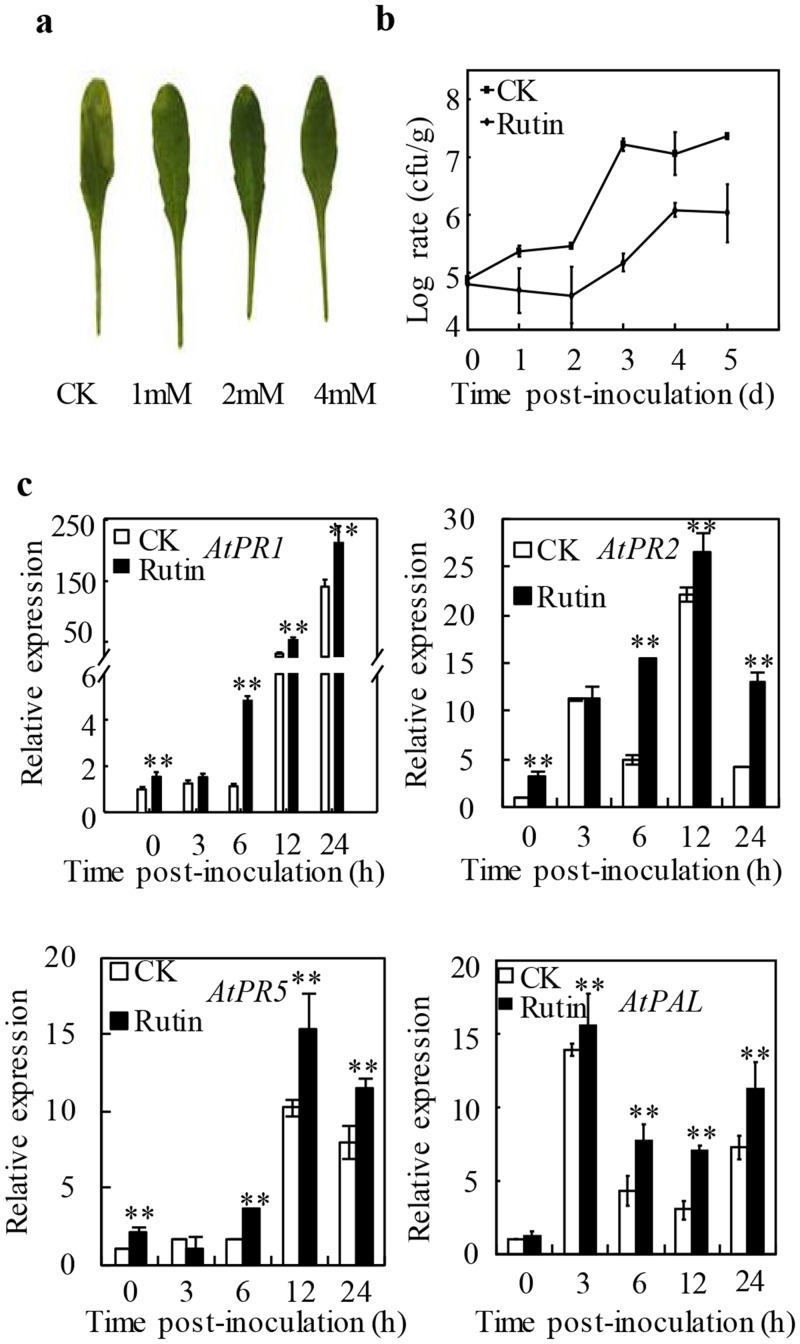
Primed the resistance against Pst DC3000 in *Arabidopsis thaliana* (Col-0). **a** Typical necrotic lesions normally caused on Arabidopsis ecotype Col-0 by the Arabidopsis pathogen *Pst* DC3000 were suppressed in rutin-treated plants. The photographs were taken 3 days post inoculation; **b** The growth of *Pst* DC3000 in control and in rutin-pretreated *Arabidopsis thaliana* (Col-0) plants. Samples were collected from 10 plants at 0, 1, 2, 3, 4 and 5 days post inoculation. The values are means ±SE; **c** Relative expression levels of resistance-related genes (*PR1*, *PR2*, *PR5* and *PAL*) in control and in rutin-pretreated *Arabidopsis thaliana* (Col-0) plants. Samples were collected from 10 plants at 0, 3, 6, 12 and 24 hours post inoculation. The values are means ±SE.

In addition to the above mentioned rice-*Xoo* and *N*. *benthamiana*-*R*. *solanacearum* investigation, we also tested the function of rutin in *Arabidopsis thaliana*. The results demonstrated that rutin also protected susceptible *Arabidopsis* ecotype Columbia-0 (Col-0) against the virulent *Pseudomonas syringae* pv. *tomato* strain DC3000 (*Pst* DC3000). After inoculation with *Pst* DC3000, typical wilting and chlorotic symptoms was observed on the leaves without pre-spraying with rutin at 3 dpi. However, attenuated disease symptoms were observed on *Arabidopsis* leaves pre-sprayed with 1, 2 and 4 mM rutin (Fig 4a). The proliferation of *Pst* DC3000 was indicated that the growth had been inhibited in leaves which were pretreated wtih 2 mM rutin (Fig 4b). More than 111.78 folds of *Pst* DC3000 had been identified in control leaves.

To understand the mechanisms involved in rutin-mediated resistance in *Arabidopsis*, we analyzed the expression patterns of four *PR* genes including *AtPR1*, *AtPR2*, *AtPR5*, and *AtPAL*, which are involved in defense responses to pathogen attack (Fig 4c). Similar with our observation in *N*. *benthamiana*-*R*. *solanacearum* and rice-*Xoo* interactions, four *PR* genes was shown more rapidly and stronger expressing activation in rutin-pretreated plants than in control plants after inoculation with *Pst* DC3000 (Fig 4c). These results suggested that rutin had the function of primed resistance in a broadly range of host, including *Arabidopsis*.

### Rutin-mediated priming is dependent on the SA signal pathway in *Arabidopsis*

**Fig 5:**
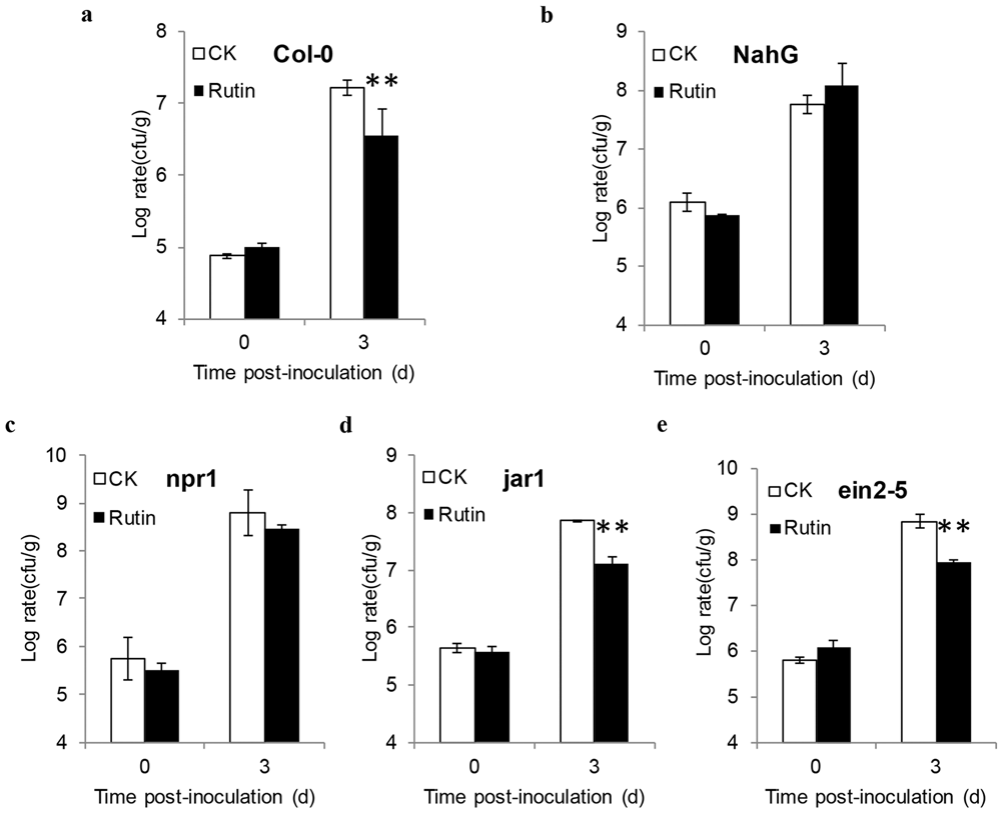
Analysis of rutin-primed resistance in *Arabidopsis* mutants. **a-e** represent as Wild type, NahG, *npr1*, *jar1*, and *ein2-5*, respectively. The growth rate of *Pseudomonas syringae* pv *tomato* strain DC3000 (*Pst* DC3000) on *Arabidopsis* mutants leaves, which was measured at 0, 3 days post infiltration. Samples were collected from 10 plants. The results show average values of triple experiments with similar results. The values are means ±SE. The asterisks denote significant differences (t -test, P < 0.01).

Plant hormones were known as the signals of plant defense. To explore the resistance signal transduction pathway mediated by rutin, a set of *Arabidopsis* mutants were used for investigation which was involved in SA, JA and ethylene (ET) dependent pathway. The *NahG* transgenic plant abolishes the accumulation of SA, and the *Arabidopsis* mutant *npr1* was a typical mutant of SA-dependent pathway. *jar1-1* and *ein2-5* were typical mutants of JA- and ET- dependent pathway. If the rutin-mediated priming defense is dependent on one of them, the inhibition growth of *Pst* DC3000 by pretreatment with 2 mM rutin will be attenuated. The results demonstrated that it was still able to inhibit the growth of *Pst* DC3000 in *jar1-1* and in *ein2-5*, while not in *npr1-1* and *NahG* plants (Fig 5). It was indicated that the rutin-mediated plant resistance is dependent on the SA signal pathway in *Arabidopsis* and independent on the JA and ET pathways.

### Rutin-mediated signaling initiated from upstream of NDR1, PAD4 and EDS1

**Fig 6:**
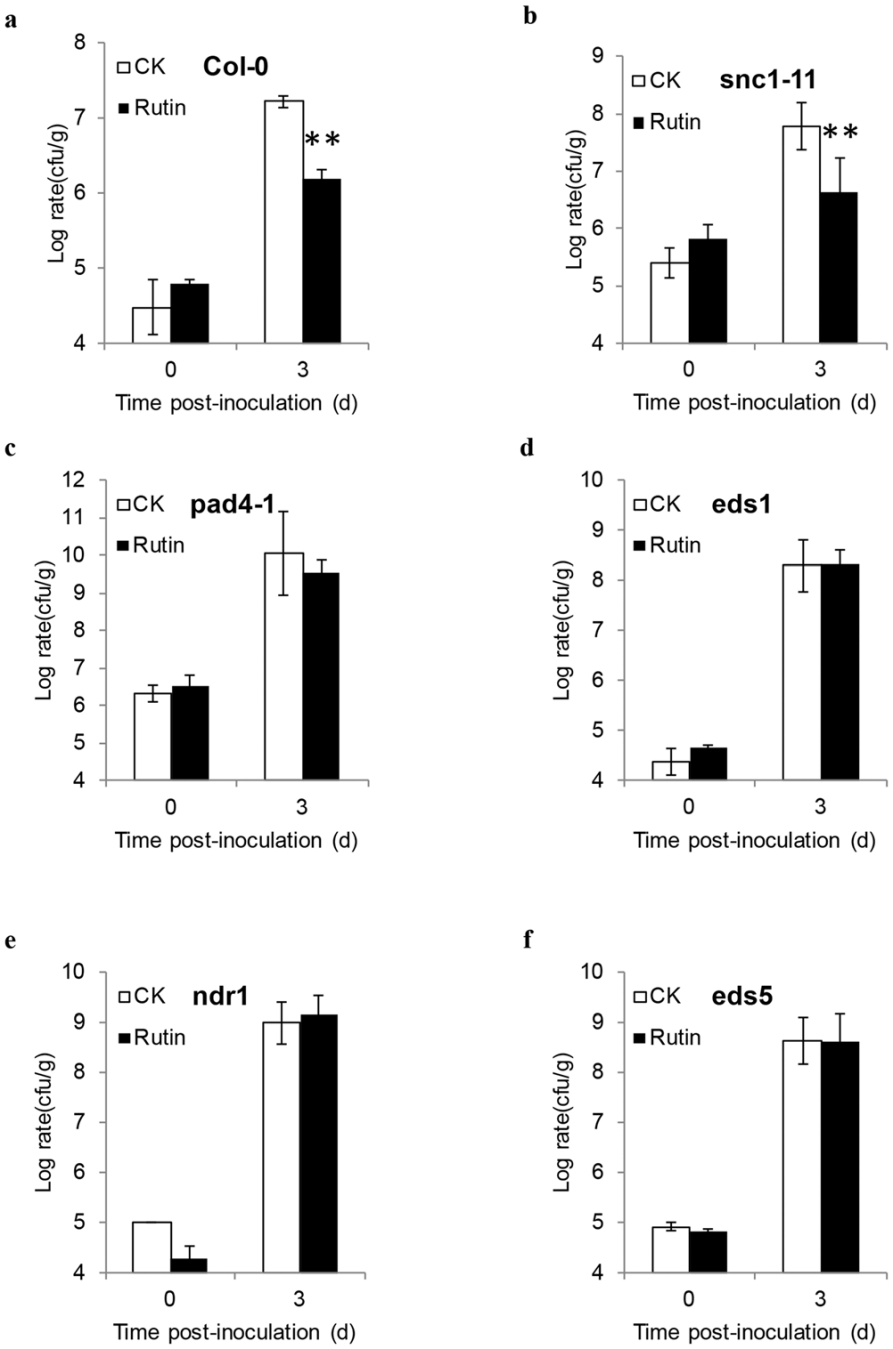
Analysis of rutin-primed resistance in Arabidopsis mutants. **a**-**f** represent as Wild type, *eds5*, *snc1-11*, *pad4-1*, *eds1*, and *ndr1* respectively. The growth rate of *Pseudomonas syringae* pv *tomato* strain DC3000 was measured. In total, samples from control and treated plants were measured at 0, 3 days post inoculation. The data were collected from 10 plants. The data showed representative experiments that were repeated three times. The values are means ±SE. The asterisks denote significant differences (t -test, P < 0.01).

To obtain more details about the signals of rutin-mediated resistance, we had investigated the growth of *Pst* DC3000 in several other mutants involved in SA signaling pathway, including with *snc1-11*, *pad4-1*, *ndr1*, *eds5* and *eds1* [29]. *SNC1* is encoded an interleukin-1 receptor-like nucleotide-binding site leucine-rich repeat type of resistance (R)-like gene residing in the *RPP5* gene cluster which possibly mediates race-specific disease resistance [30–32]. EDS1 and PAD4 belong to two lipase-like proteins [33], NDR1 is a putative membrane-binding protein [34] and EDS5 is an MATE-like SA transporter which pumped the SA from the chloroplast to the cytoplasm [35]. These proteins belong to three upstream components responsible for the transduction of SA signals and for downstream pathways triggered by the R protein. Except in *snc1-11*, the inhibition growth of *Pst* DC3000 by pretreatment with 2 mM rutin had been attenuated in *pad4-1*, *ndr1*, *eds 5* and *eds1* (Fig 6). These results were further suggested that the rutin-mediated resistance was dependent on the SA signal pathway, which was initiated upstream of NDR1, PAD4 and EDS1.

## Discussion

Rutin, classified as a polyphenolic substance, had also shown to exhibit bactericidal and fungicidal activity *in vitro* assay. The antibacterial activity of rutin was reported to specific bacteria species, such as *Xanthomonas campestris*, *Agrobacterium tumefaciens*, *Xylella fastidiosa* etc [19, 20]. The possible mechanism of action is presumably as follows: first, such polyphenolic substances most likely disrupt the cell wall and the cell membrane integrity of microbial cells, which leads to the release of intracellular components and causes the electron transfer at the membrane, the repression of nucleotide synthesis and ATP activity, thereby inhibiting the growth of microorganisms [36]; Second, rutin excessively scavenges the reactive oxygen species of microbes, leading to a reduction in the normal physiological function of reactive oxygen [37]. But rutin were effective in inhabiting bacteria causing the plant disease at relative high minimum inhibitory activity (MIC) which means to weaker bactericidal activity than other phenolic compounds [19, 20]. In this study, we have measured the inhibition efficiency of rutin against four plant bacterial pathogens, including *R*. *solanacearum* SD, *Xanthomonas oryzae* pv. *oryzae* (PXO99), *Xanthomonas oryzae* pv. *oryzicola* (RH3) and *Pst* DC3000. The results demonstrated that rutin was only functional in very high concentration over 4 mM (Fig 1). Our other study demonstrated that the AtMYB12-overexpressing tobacco had approximately enriched the averaged concentration of rutin as 1.43 mM in fresh weight. It was also enhanced resistance against *R*. *solanacearum* (Li et al., unpublished data). Together, the conclusions of this work were consistent with previous studies that rutin has demonstrated weak antibacterial activity to against three additional species of plant gram-negative bacterial pathogens.

*In vitro* assays, 2 mM rutin hardly inhibit the growth of *R*. *solanacearum* SD, PXO99 and *Pst* DC3000 in medium. Causing we hardly quantify the concentration of rutin for intercellular space, we couldn’t completely eliminate the direct inhibition caused by the antibacterial agent. However, spraying 2 mM of rutin dramatically reduced the growth of those bacteria in each host plant, which implied that other resistance mechanisms had been triggered (Figs 2 and 3). Notably, the foliar application of 2 mM rutin almost rarely affected the expression of the SA-responsive *PR1a* gene on *N*. *benthamiana* (Fig 2c). This was indicated that rutin couldn’t directly activate the basal plant defense. Interestingly, when challenged with a pathogen, the plants pre-spraying rutin show a faster and stronger expression of *PR1a* than control as well as other *PR* genes (Figs 2d, 3d and 4c). The delay of occurrence resistance was indicated that rutin promotes disease resistance by a priming mechanism. In addition, exogenous application of rutin simultaneously enhanced the expression of genes which involved into SA, reactive oxygen species and nitric oxide signal pathway (Figs 2 and 3), indicating the ownstream signaling activated by rutin was complex.

**Fig 7:**
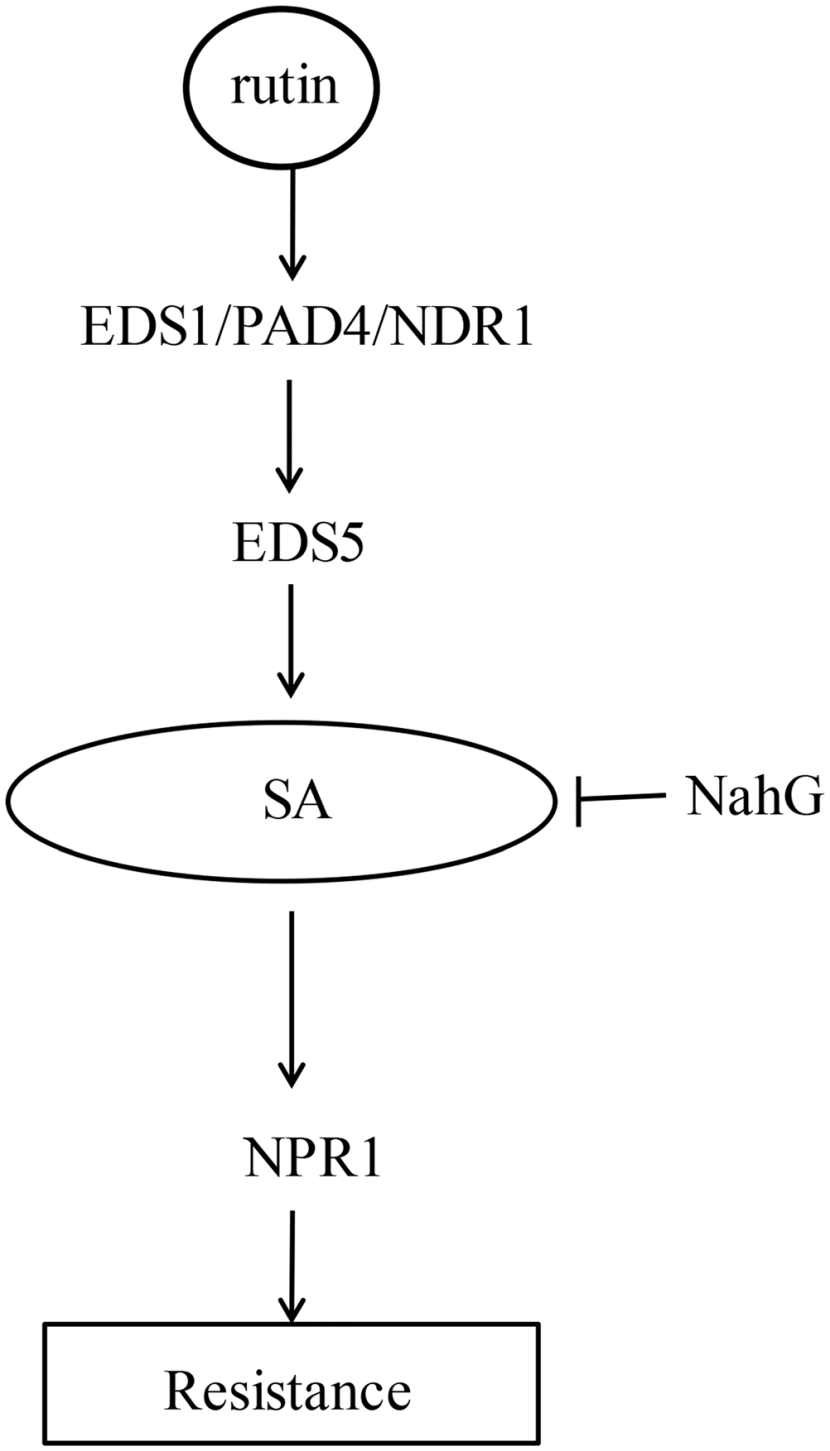
Working model for rutin-primed signal transduction pathway. The components that are relevant to this study are shown. The resistance signal activated by rutin is located upstream of NDR1, PAD4 and EDS1, and probably specifically affect R proteins or some downstream components of the SNC1 and other R protein. Rutin-primed plant resistance was compromised in the defective SA pathway mutants (*NahG*, *npr1*, *eds5*), Arrows point to event fluids.

Many chemicals or plant metabolic components have also been reported to induce or prime plant defense responses that are dependent on the SA signal transduction pathway. However, most of these studies primarily focused on the characterization of the effects of these components using *NahG* and *npr1* mutants [12,13,15], except for azelaic acid which has been analyzed to induce plant defense responses dependent on NDR1 and PAD4, which are two importance components involved in the upstream signals of SA [14]. Rutin-stimulated plant resistance was compromised in many defective SA pathway mutants, confirming that SA signaling was required for rutin-primed disease resistance (Figs 5 and 6). Our data have also identified that NDR1, PAD4 and EDS1 were required for rutin-primed plant defense (Fig 6). This result implied that rutin-primed plant resistance might slightly differ from other plant activators. NDR1 and EDS1 mediated the signal downstream from the major subsets of R proteins, including the CC-NBS-LRR type and the TIR-NBS-LRR type, and they represent an important node acting upstream of SA in PTI [38, 39]. Interestingly, we have determined that *snc1-11* mutant did not affect the rutin-primed resistance, given the possibility that rutin may specifically affect the other R proteins or downstream components of the SNC1 and other R protein. Based on these results, the possible work model for rutin-primed defense is described (Fig 7), which temporally suggests that the resistance signal is initiated upstream from NDR1, PAD4 and EDS1, followed by activating SA signal transduction. Even though we did not decipher the beginning signals or the targeted receptor of rutin in plants, nevertheless, this study it still offers a new research insight into this newly characterized plant activator.

Flavonoids play a critical role in preventing human diseases and have been evolved as a protective mechanism for different plants. In this study, we also found that rutin as a component of flavonoids which could involve into plant immunity with a broad range of host. Together with the quercetin [22], it is feasible suggestion of a conserve mechanism for priming the plant immunity with other components of flavonoids. As rutin was functional at a relative high concentration and economical cost, it is formidable to use directly as a purify bactericide. However, there has increasingly growing of reports that the high content of rutin could be regulated synthesis and accumulated in plant by several transcriptional factors, including with *AtMYB11*, *AtMYB12* and *AtMYB111* [23, 40–42]. It was provided opportunity to promote the use of rutin by reducing the economic cost in future. Additionally, AtMYB12-expression tobacco was also reported to be resistant to insects, such as aphid, whitefly, *Spodoptera litura* and *Helicoverpa armigera*, by the high-level accumulation of rutin [23, 40]. And our previous study showed that the flavonol enriched *AtMYB12*-expression tobacco enhanced the resistance against pathogens, such as *R*. *solanacearum*, *Colletotrichum nicotianae Averna* and *Alternaria alternata*. The priming resistance identified with rutin would be helpful to understand the resistance generated by *AtMYB12*-expression tobacco. It was opens the opportunity to make the daily nutrient and biosafety bactericide with overcapacity simultaneously by transgenic method.

## Supporting Information

S1 Fig The influence of different concentration of quercetin on bacterial pathogens. The growth condition of various bacterial on the plate with different concentration of quercetin. The photographs were taken after 24 h incubation at 28°C.
(TIF)

S2 Fig The comparison of anti-bacterial activity of rutin and quercetin. **a**The growth curve of *R*. *solanacearum* SD in *N*. *benthamiana* after 2mM rutin and 1mM quercetin pretreatment. The data represent the mean ±SE of 5 plants. **b** The lesion length causing by *Xanthomonas oryzae* strain PXO99 after 2mM rutin and 1mM quercetin pretreatment. The data were collected at 7 and 14 days post inoculation. The data represent the mean ±SE of 5 plants.
(TIF)
