# Supplementary figures and images for: Rutin-Mediated Priming of Plant Resistance to Three Bacterial Pathogens Initiating the Early SA Signal Pathway
Source: PLoS One. 2016 Jan 11;11(1):e0146910. doi: 10.1371/journal.pone.0146910 (PMC4713477; doi:10.1371/journal.pone.0146910)

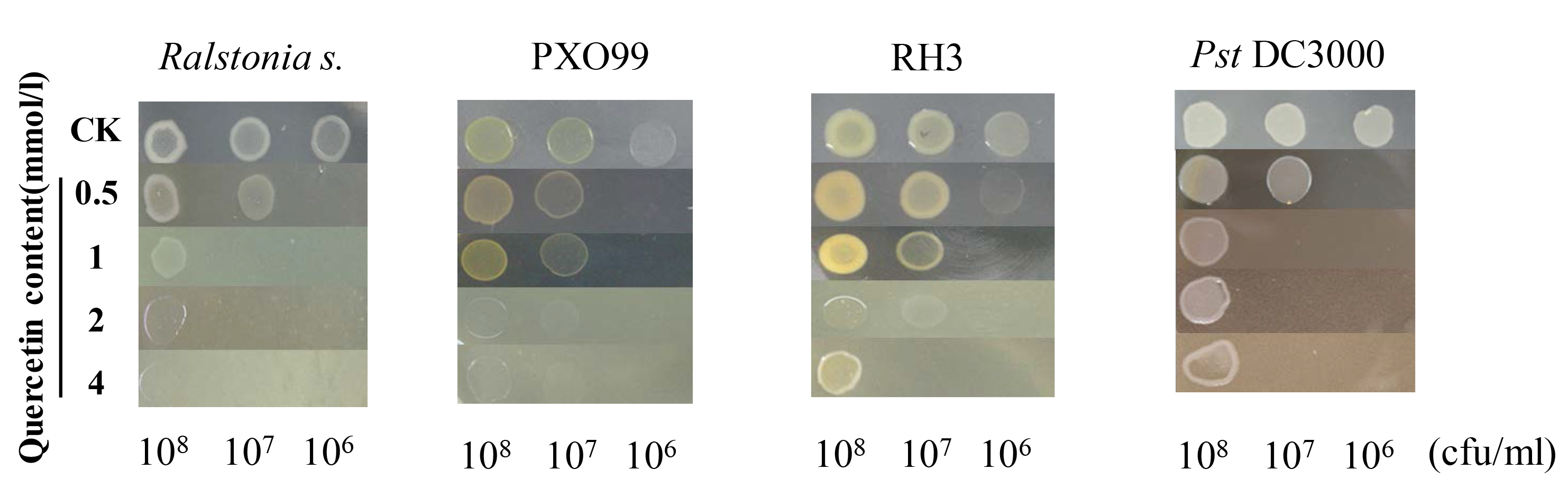

Supplement: S1 Fig — The growth condition of various bacterial on the plate with different concentration of quercetin. The photographs were taken after 24 h incubation at 28°C. (TIF) [file pone.0146910.s001.tif]

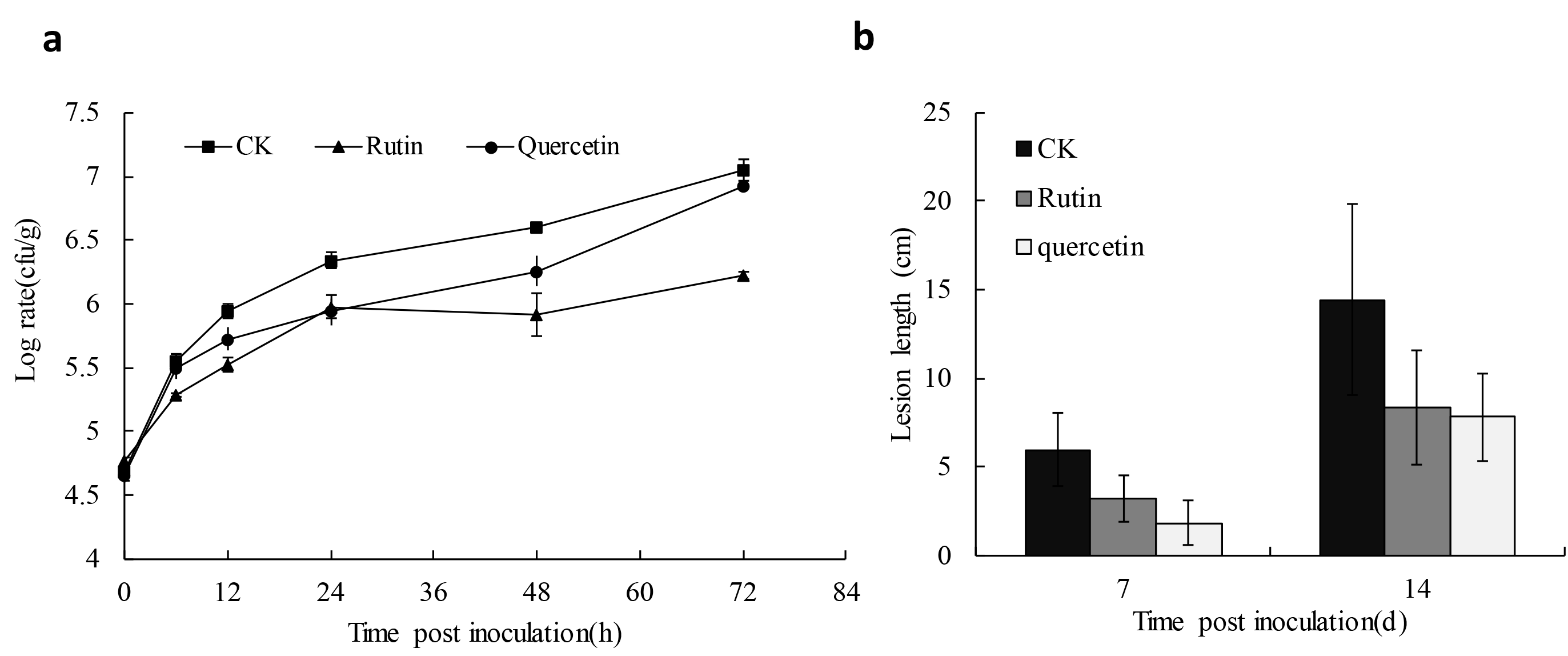

Supplement: S2 Fig — aThe growth curve of R. solanacearum SD in N. benthamiana after 2mM rutin and 1mM quercetin pretreatment. The data represent the mean ±SE of 5 plants. b The lesion length causing by Xanthomonas oryzae strain PXO99 after 2mM rutin and 1mM quercetin pretreatment. The data were collected at 7 and 14 days post inoculation. The data represent the mean ±SE of 5 plants. (TIF) [file pone.0146910.s002.tif]
